# Examination and Comparison of Cognitive and Executive Functions in Clinically Stable Schizophrenia Disorder, Bipolar Disorder, and Major Depressive Disorder

**DOI:** 10.1155/2020/2543541

**Published:** 2020-12-14

**Authors:** Behrooz Afshari, Nasrin Shiri, Fatemeh Sadat Ghoreishi, Mohtasham Valianpour

**Affiliations:** ^1^Kashan University of Medical Science, Kashan, Iran; ^2^Islamic Azad University-Kashan Branch, Kashan, Iran

## Abstract

**Background:**

Schizophrenia (SC), bipolar disorder (BD), and major depressive disorder (MDD) are associated with various cognitive and executive dysfunctions. The aim of the present study was to evaluate and compare cognitive and executive dysfunctions in schizophrenia, bipolar disorder, and major depressive disorder.

**Materials and Methods:**

Sixty-four schizophrenia patients, 68 bipolar patients, 62 patients with major depressive disorder, and 75 healthy individuals participated in the present study. All participants were assessed with the Structured Clinical Interview for DSM-IV Axis I Disorders (SCID-I), Young Mania Rating Scale (YMRS), Positive and Negative Syndrome Scale (PANSS), Beck Depression Inventory (BDI-II), Trial Making Test (TMT), Four-Choice Reaction Time Task, Ruler Drop Method (RDM), Tower of London (TOL) task, and the Wisconsin Card Sorting Task (WCST). Data were analyzed by chi-square, Kolmogorov-Smirnov, and independent *t*-tests; ANOVA; and MANOVA.

**Results:**

In the cognitive function, the scores of SC, BD, and MDD patients were lower than those of healthy individuals. Also, the scores of MDD patients were lower than those of other patients, and the scores of BD patients were lower than those of SC patients. In the executive function, the scores of SC, BD, and MDD patients were lower than those of healthy individuals. Moreover, the scores of the MDD group were higher than those of the BD and SC groups, and the scores of the SC group were higher than those of the BD group.

**Conclusion:**

Patients with SC, BD, and MDD have poorer cognitive and executive functions than healthy individuals, even when these patients are in a stable state. Assessment of cognitive and executive functions in SC, BD, and MDD patients can help in understanding the pathology of these disorders.

## 1. Introduction

Schizophrenia (SC), bipolar disorder (BD), and major depressive disorder (MDD) are associated with various cognitive and executive dysfunctions. Schizophrenia is a mental disorder whose symptoms include a wide range of cognitive, behavioral, and emotional disorders [1]. Cognitive and executive deficits are recognized as major deficits in schizophrenia disorder and are highly correlated with functional deficits. Schizophrenia patients exhibit a wide range of cognitive and executive deficits including attention, response inhibition, cognitive flexibility, and processing speed [2].

Gold et al. (2002) reported that semantic fluency, verbal memory, and processing speed can predict work continuity in schizophrenia. Daily problem-solving skills also depend on verbal memory, verbal fluency, and processing speed [3].

McLaren et al. (2007), Van Beilen et al. (2004), and Henry and Grawford (2005) concluded that decelerating processing speed is associated with impaired function in SC patients [4, 5]. Their findings suggest that processing speed is an important predictor of SC patients' performance [6].

Evidence suggests that information processing speed in patients with SC is low. Also, memory deficits may contribute to the slow processing speed of these patients [7]. Some studies have also reported a relationship between cognitive and functional deficits with negative symptoms in patients with SC [8].

In addition to poor cognitive function, executive dysfunction in the SC is also impaired. Deficits associated with frontal cortex are known to be responsible for the severity of clinical symptoms and poor executive function, such as cognitive inflexibility and working memory deficits in SC disorder [9]. Neuropsychological studies using the Wisconsin Card Sorting Task have shown executive dysfunction in both the first episode of SC and chronic SC. Poor performance in patients with SC is associated with poor cognitive flexibility, problem solving, and attention control in the Wisconsin Card Sorting Task. Executive dysfunction predicts poor performance in patients, failure in therapeutic interventions, and limited rehabilitation [10]. One of the important questions raised in the field of SC is whether the neuropsychological deficits in SC are more severe than other disorders such as bipolar disorder and major depressive disorder.

Studies showed that BD also has deficits in attention, executive function, cognitive function, cognitive flexibility, and memory [11]. In BD patients, reaction time delay is observed in emotional situations whereas this delay is not observed in normal situations [6]. Processing speed is the cornerstone of many cognitive processes. Many studies have shown that cognitive processing speeds in bipolar patients are slower than normal [12]. The findings of a study showed that patients with BD have cognitive dysfunction in processing speed and visual memory, which may be a genetic feature of BD patients [13]. Bipolar disorder is also associated with deficits in executive functions such as cognitive flexibility and attention change [14]. Also, performance-related deficits are associated with the number of mood swings in bipolar disorder [15]. Some studies showed that the range of cognitive and executive dysfunction in BD patients is related to the course of illness and length of hospitalization, and BD patient deficits in the mania episode are more extensive than in other episodes [16].

Many studies on MDD also showed widespread functional and cognitive deficits in these patients [17, 18]. Major cognitive deficits in MDD include poor processing speed and poor verbal memory [19]. Cognitive deficits in MDD patients are also associated with the severity of clinical symptoms. Some theoretical models have suggested that cognitive deficits can be a risk factor for future depression [18]. Neuropsychological tests in MDD patients have shown poor performance in these patients. Impaired executive function in MDD patients is characterized by impairment in processing speed, impaired vision, and behavioral disability [20].

Although the cognitive and executive deficits of BD patients are apparently lower than those of SC, the cognitive profiles of these two disorders are similar. However, cognitive deficits in these patients may follow different pathways [21]. Some studies showed that these disorders are more severe in SC patients. Krabbendam et al. showed that cognitive performance in SC patients was significantly worse than in bipolar patients [11]. Working memory, short-term verbal memory, and long-term verbal memory in SC are weaker than BD. Some studies have reported similar deficits in SC and BD, while other studies reported more severe deficits in SC [22]. Although some studies have reported similar executive dysfunctions in the BD and SC patients, other studies have considered the executive dysfunction of bipolar patients between SC patients and healthy individuals [23]. Given the above findings, many studies have shown a link between cognitive and executive dysfunctions and the prognosis and symptoms of SC, bipolar disorder, and MDD. Also, assessment of cognitive and executive dysfunction in SC, BD, and MDD patients can help to understand the pathology of these disorders. Therefore, the aim of the present study was to evaluate and compare cognitive and executive functions in SC, BD, and MDD.

## 2. Materials and Methods

### 2.1. Participants

#### 2.1.1. Demographic Characteristics

A total of 269 participants across four groups (SC *n* = 64, BP *n* = 68, MDD *n* = 62, and HC *n* = 75) were included in this study. Included in the SC group were 32 women (50%), aged between 18 and 44 years. In the BD group, 38 were women (55.9%), aged between 22 and 45 years. In the MDD group, 37 were women (59.7%) who were aged between 23 and 43 years. The HC group consisted of 46 women (61.33%), aged between 18 and 45 years. Characteristics of the study sample are presented in Table 1.

#### 2.1.2. Clinical Characteristics

All clinical participants were taking medication. The most commonly used medications for the SC group were Clonazepam, Depakine, Clozapine, and Quetiapine; for the BD group, they were lithium, SSRIs, Clozapine, and Carbamazepine; and for the MDD group, they were SSRIs, SNRIs, TCAs, and MAOIs. The HC group was not on medication because that was a sample of healthy people with no mental disorders. In addition to meeting the diagnosis of SC, BD, or MDD, 29.58% of the SC group, 36.76% of the BD group, and 34.65% of the MDD group meet the diagnostic criteria for at least one other disorder. Specific clinical characteristics were measured for the SC and BD groups. For the SC group, the negative symptom scale of PANSS (*M* = 24.02, SD = 4.08) and the positive symptom scale (*M* = 11.09, SD = 3.06) were used. For the BD group, the YMRS (*M* = 13.06, SD = 7.04) was used. Means and standard deviations of demographic and clinical characteristics are presented in Table 1.

### 2.2. Procedure

Ethical approval from the Research Deputy of University was received prior to recruitment. Clinical groups were recruited from the hospital (a psychiatric hospital located in Iran) and six different mental health clinics, in Iran, between February 2018 and April 2019. Participants in the clinical groups were referred to the study from the hospital and mental health clinics. After providing informed consent, a mental health assessment was made by a trained psychiatrist using a structured clinical interview for DSM-IV Axis I disorders [24]. Participants provide demographics and cognitive tests at the same time as this mental health status assessment. The data were not from medical records, and we collected them. The HC group was recruited from the general population and was invited to take part in the study by researchers.

For inclusion in the clinical groups, participants needed to meet the DSM-IV criteria for SC, BD, or MDD and have at least eight years of formal education—this was needed to ensure that the tasks and questionnaires were understood. Participants were screened for inclusion by a trained psychiatrist using the SCID-I during an interview arranged with the study psychiatrist at the University. Exclusion criteria for clinical groups were current mania, psychotic episodes, and severe depression. Because the aim of the present study was to assess clinically stable patients, we exclude patients with severe symptoms. Participants in the HC group were included if they did not meet the criteria for any current axis I disorder of DSM-IV. After obtaining informed consent, SCID-I, BDI-II, SGIT, PANASS, YMRS, TMT, Four-Choice, RDM, TOL, and WCST were administered to the four groups.

### 2.3. Measure

#### 2.3.1. Demographic Characteristics and Premature Intelligence (SGIT)

Demographic characteristics include age, gender, education level, age of disorder diagnosis, number of hospitalization, comorbidity with other mental disorders, and the use of medication. Also, the patient's predictive intelligence was determined using SGIT. The SGIT assessed general intelligence in the four groups [25].

#### 2.3.2. Structured Clinical Interview for DSM-IV Axis I Disorders (SCID-I)

The SCID-I is one of the most widely used structured interviews for the presence of axis I disorders, including schizophrenia and bipolar disorder. The SCID-I has a screening form consisting of 24 items evaluating symptomology for various axis I disorders [24, 26]. The SCID-I has demonstrated appropriate psychometric characteristics in the Iranian (Persian) community. Diagnostic agreements between test and retest SCID-I administration were fair to good for most diagnostic categories. Overall weighted kappa was 0.52 for current diagnoses and 0.55 for lifetime diagnoses. In an Iranian healthy sample, the internal consistency of SCID-I using Cronbach's alpha ranged from 0.66 to 0.79 [27]. Specificity values for most psychiatric disorders were high (>0.85); the sensitivity values were somewhat lower [28].

#### 2.3.3. Young Mania Rating Scale (YMRS)

The YMRS measures mania symptom severity. This 11-item scale has 7 items scored from 0 to 4 and 4 items from 0 to 8, completed after a clinical interview with the patient. The YMRS [29] is a tool with validity, sensitivity, and specificity and is suitable for clinical and research work. The YMRS showed a reliability index of 0.88 (internal consistency) and 0.76 (test-retest reliability) when compared to the mania subscale of the Modified Clinical Global Impression [30, 31]. YMRS had a high internal consistency (Cronbach′s alpha = 0.82) in the Iranian population, and it also showed a high concurrent validity with a mixed subscale of BDRS (*r* = 0.745) [32, 33].

#### 2.3.4. Positive and Negative Syndrome Scale (PANSS)

The Positive and Negative Syndrome Scale (PANSS) was designed to measure the severity of the positive and negative symptoms of schizophrenia patients. Positive symptoms are delusions, hallucinations, disorganized speech, and grossly disorganized or catatonic behavior. Negative symptoms are social withdrawal, difficulty paying attention, apathy, and affective flattening. The questionnaire has 30 questions, and the subject answers it using a seven-choice scale [34, 35].

#### 2.3.5. Beck Depression Inventory (BDI-II)

The BDI-II is a 21-item self-report questionnaire designed to assess depression. The scores range from 0 to 63, with a score of 10-19 considered as mild depression, a score of 20-28 denoting medium depression, and a score of 29-63 as severe depression [36]. In an Iranian healthy sample, the internal consistency of BDI-II using Cronbach's alpha ranged from 0.58 to 0.79 for all factors [37]. Internal stability of the test on Iranian students was estimated as 0.87 and its reliability was 0.73 (38).

#### 2.3.6. Trial Making Test (TMT)

In this test, the subject first connects numbered circles sequentially by drawing a line between them (Section A), and then in the next form, the numbered ranges are assigned to the numbers marked with the connected letters (Section B). Scoring is based on the total time taken to complete Form A and Form B. In Section A, the time less than 20 seconds is normal, and the time greater than 78 seconds is with a defect in the visual processing speed. In Section B, the time of 75 seconds is normal, and the time greater than 273 seconds is with a defect in the visual processing speed. This test is based on two factors: the “visual-space chain” and “visual quick-search” saturation [38].

#### 2.3.7. Four-Choice Reaction Time Task

The task was programmed in a computer screen. Participants were seated in front of a touch-screen computer and were instructed to press the space bar with the index finger of their dominant hand. Participants released the space bar and touched the box on the screen in which the target had appeared. They were asked to respond as quickly as possible. The accuracy was measured as a ratio of correct responses divided by the sum of correct and incorrect responses. The trials in which the subject touched the wrong box after the target onset were defined as incorrect responses [39].

#### 2.3.8. Ruler Drop Method (RDM)

RDM is a reaction time test that is assessed by a ruler. In this research, a 30 cm ruler was used. In this test, the lower score indicates lower reaction time and better performance [40, 41].

#### 2.3.9. Tower of London (TOL) Task

The TOL is an executive function task originally developed by Tim Shallice, with the purpose of planning goal-directed behaviors, working memory, and inhibition abilities. This task consists of three balls of differing colors. There are two sets of three colored balls present; one in the upper half of the screen and one in the lower half. In each trial, the red, blue, and green balls of both sets are shown in predetermined but different positions. The TOL is mainly used as a planning tool in a variety of domains. The TOL has been widely used in clinical individuals in order to assess the planning abilities and frontal lobe functions [42, 43]. In an Iranian healthy sample, the internal consistency of TOL using Cronbach's alpha was 0.72 to 0.78 [44]. This task was presented and completed on a computer (PEBL software).

#### 2.3.10. The Wisconsin Card Sorting Task (WCST)

The WCST incorporates four types of stimulus cards that vary by number, shapes, and color. This task evaluates problem solving, task-switching, inhibition, planning, and working memory and is a measure of an individual's executive function skills [45]. In this computer task, subjects are classified into 128 cards using four templates. The WCST main indicators are the number of correct responses, total errors, number of continues reaction, perseverative errors, nonperseverative errors, and the number of completed categories [29]. In an Iranian healthy sample, the internal consistency of WCST using Cronbach's alpha was 0.73 to 0.74 [46]. This executive function task requires participants to sort cards according to a rule that is not made clear to them, and so they must discover the rule using trial and error. This task was presented and completed on a computer (PEBL software).

### 2.4. Data Analysis

Measures of executive and cognitive functions were standardized using means and standard deviations from the full baseline sample. Analysis of variance (ANOVAs) and the chi-square test were used to examine baseline characteristics of participants. Also, Kolmogorov-Smirnov and the independent *t*-tests were used to compare demographic and clinical variables in the four groups. In this research, MANOVA was employed for the three subscales on WCST and the two subscales on TMT.

## 3. Results

Age of participants was similar in four groups (*F*_(2, 207)_ = 18.1, *p* = 0.687). Patients with schizophrenia had significantly more depressive symptoms (*F*_(7, 216)_ = 82.5, *p* = 0.001; see Table 1). In the SC group, the number of women and men were equal. In the BD and HC groups, there were more women than men, but this difference was not statistically significant (*χ*^2^(2) = 1.85, *p* = 0.362).

The mean age of the BD group (*F*_(2, 207)_ = 18.1, *p* = 0.687) and level of education of the HC group (*F*_(3, 124)_ = 24.5, *p* = 0.142) were higher than those of the other two groups. Also, these differences were not statistically significant. The age of the first hospitalization was lower in the SC group (*t* = 2.1, *p* = 0.548). The history of hospitalization number mean (*t* = 0.3, *p* = 0.654) and comorbidity with other disorders (*χ*^2^(2) = 1.1, *p* = 0.027) were higher in the BD group.  Table 2 presents the cognitive and executive function scales.

The mean, standard deviation, significance, and effect size of the four subscales of cognitive and executive function among the three groups are shown in Table 2.

The results of MANOVA showed a significant difference between the SC, BD, MDD, and HC in terms of their scores on Carver-White's cognitive and executive function scales (Pillai′s trace = 0.659; *F*_(8,306)_ = 18.79; *p* < 0.001; partial *η*^2^ = 0.329). MANOVA showed significant differences between the four groups in all scales of cognitive and executive function. The results are shown in Table 2.

### 3.1. Cognitive Function Results

The scores of the MDD group in SGIT were higher than those in the SC and BD groups but lower than those in the HC group (*p* ≤ 0.001). Also, MDD, BD, and SC groups were lower on TMT a, TMT b, Ruler, and RT time than the control group. The scores of the MDD group were lower than those of the SC and BD groups, and also, the scores of the BD group were lower than those of the SC group on these scales (*p* ≤ 0.001). For RT accuracy, the score of the control group was higher than those of the other groups; the score of the MDD group was higher than those of the BD and SC groups; also, the score of the BD group was higher than that of the SC group (*p* ≤ 0.001). Cognitive function scales are shown in Table 2.

### 3.2. Executive Function Results

The scores of the HC group in TOL were higher than those in the clinical groups. Moreover, the TOL score was more frequent in the MDD, SC, and BD groups. Correct responses in WCST were higher than those in the MDD, BD, and SC groups. The scores of the MDD group were higher than those of the BD and SC groups, and the scores of the SC group were higher than those of the BD group on these subscales. Furthermore, more frequent nonperseverative errors were found in the SC group (*N*: 49, *p* ≤ 0.001), MDD group (*N*: 48, *p* ≤ 0.001), and BD group (*N*: 39, *p* ≤ 0.001). Also, maximum perseverative errors were in the BD group (*N*: 40, *p* ≤ 0.001), SC group (*N*: 9, *p* ≤ 0.001), and MDD group (*N*: 8, *p* ≤ 0.001). Executive function scales are shown in Table 2.

## 4. Discussion

Cognitive and executive dysfunctions are major components of SC, BD, and MDD [47]. The purpose of the present study was to evaluate and compare cognitive and executive dysfunctions in SC, BD, and MDD. One of the major differences of this study from previous studies is that in this study, patients with SC, BD, and MDD were under medication and in stable condition. Cognitive functions in the present study included general intelligence, cognitive speed, and reaction and decision time. Also, although executive dysfunctions include planning, task-switching, cognitive flexibility, inhibition, problem solving, working memory, and several other subcomponents, our aim was to evaluate planning, problem solving, and cognitive flexibility. The results of the present study showed that patients with SC, BD, and MDD performed poorly in cognitive and executive functions than healthy individuals, even when patients are in the maintenance and stable state. Cognitive deficits are widely observed in SC [48], BD [49], and MDD [20]. Cognitive and executive functions are associated with the severity of symptoms in SC. In SC patients, more cognitive deficits are associated with longer disease duration and more negative symptoms [8].

Many previous studies have shown that cognitive deficits in SC patients are more than those in BD patients [50, 51]. However, the results of the present study show that cognitive deficits in BD patients are more than those in SC patients, and this finding is in line with some previous research in comparing cognitive symptoms in SC and BD patients [11, 52]. Some studies showed that the range of the cognitive and executive dysfunctions in BD patients is related to the episode of illness and length of hospitalization, and BD patient deficits in the mania period are more extensive than in others. In BD patients, higher levels of cognitive deficits are associated with frequent hospitalizations, behavioral, emotional, and cognitive responses as well as the inability to delay rewards. These deficits are also more severe in manic episodes of BD disorder than in other episodes of the disorder [16].

Although the mechanism underlying cognitive deficits in individuals is unclear, many researchers have pointed to the fundamental effects of genes on brain function and cognitive deficits [53–55]. It is not yet clear what structural and functional differences exist in the different brain regions of patients with SC, BD, and depression. Network connectivity changes may be related to poorer performance in BD patients [56].

In the present study, although general intelligence in depressed patients is lower than that in healthy subjects, it is significantly higher than that in schizophrenia and bipolar patients. Also, general intelligence in the two groups of schizophrenia and bipolar patients was close to each other and was slightly higher in schizophrenia than in bipolar patients, but both schizophrenia and bipolar patients were significantly different from healthy subjects. This discrepancy can affect cognitive functions such as processing speed and reaction and decision time and may lead to impairment of these functions in schizophrenia and bipolar patients. Many studies have reported the association of general intelligence with different parts of the brain and neuropsychological deficits [57].

These deficits are associated with psychological dysfunctions in areas such as job performance, communication with family members, and life satisfaction. Also, these functions are associated with the severity of symptoms in mental disorders [8].

The processing speed test results showed that cognitive speed in schizophrenia, bipolar disorder, and depression was weaker than that in healthy controls. In the present study, cognitive velocity was lower in depressed patients than in other groups and also lower in bipolar patients than in schizophrenic patients and healthy controls. Cognitive speed is one of the domains of cognitive functioning that is affected by mental disorders [58, 59]. Poor processing speed is generally associated with worse pathology severity and worse social and occupational function [57]. Also, low processing speed can increase the risk of anxiety and depression [60]. According to the findings of the present study as well as the results of previous studies, severity of injury, social and occupational function impairment, and the risk of anxiety and depression in patients with major depression may be higher than those in schizophrenia and bipolar patients.

Results of the reaction and decision time tests showed that schizophrenic, bipolar, and depressive patients were significantly weaker at reaction and decision times than healthy controls. In the present study, reaction and decision times in depressed patients were less than those in the other groups. This finding correlates with features and symptoms of depressed patients, such as psychomotor retardation [25]. Depression can affect processing speed and reaction and decision times, and recent researches have shown that processing speed and reaction and decision times in depressed patients are slower than normal [61–63]. Also, depression rates in schizophrenia patients were in the severe range. This finding is consistent with previous research on the high prevalence of depression in schizophrenia patients [25].

Although the processing speed in schizophrenia patients is better than that in bipolar patients, this has led to erroneous responses, and the number of correct responses in schizophrenia patients is lower than that in bipolar patients, and although reaction and decision times in bipolar patients are higher and worse than those in schizophrenia patients, they are more accurate in responding. In bipolar patients, deficits in reaction time and decision making are associated with episodes of mania and depression, as well as impulsivity [64].

The results of the TOL and WCST tests showed that planning, problem solving, and cognitive flexibility were lower in schizophrenic, bipolar, and major depressive patients. Patients with depression are also better than those with schizophrenia and bipolar disorder. The frontal cortex plays an important role in planning, problem solving, and cognitive flexibility [65]. Also, executive function has a great deal to do with psychological performance in areas such as job performance, relationships with family members, and life satisfaction [66].

In addition, many patients with frontal lobe injuries have reported deficits in problem solving and planning their daily activities. One study showed that when people do planning and problem-solving tasks, their brain activity in the frontal cortex increases [67]. Some studies of mental disorders have revealed abnormalities in important parts of the brain such as the frontal cortex [68]. Neuropsychological studies using WCST to evaluate executive function have shown moderate to severe impairments in executive function in schizophrenia patients [69]. Also, numerous executive dysfunctions in bipolar disorder have been identified, notably that executive deficits have widespread and significant impacts on the lives of bipolar patients, indicating severe problems in controlling and regulating their behaviors [70]. Deficits associated with executive function in depressed patients also have negative effects on their daily living activities. Neuropsychological tests in depressed patients have shown poor performance in these patients. Impaired executive function in depressive patients is characterized by impaired vision, deficit in processing speed, and behavioral disability [20]. The impact of executive function is so great that we can refer to it as the basis of almost all of the disorders in the DSM [71]. The neuropsychological structure of executive function and its evaluation methods is different from the neuropsychological disorders present in DSM-5. The major flaw in the diagnostic criteria for neuropsychological disorders present in the DSM-5 is a misunderstanding of clinical neuroscience assessment, especially executive functioning assessment [72].

## 5. Limitations

One of the limitations of the present study is that because the patients were on medication, the effect of medication and drug dose on cognitive and executive functions of patients was not controlled. Using medication in patients with SC, BD, and MDD could affect the results; according to ethical principles, we could not exclude it, but we suggest future researches in patients with first episode of SC, BD, and MDD without medication. Furthermore, SC, BD, and MDD patients who participated in the present study had a long period of disorder. Therefore, we may not be able to generalize the results of this study to patients who have experienced a short period of their disorder. Moreover, comorbidity between schizophrenia and depression interferes with the test results. Given that the study is aimed at comparing SC, MDD, and BD populations, higher rates of comorbidity among these disorders might work as confounders when interpreting test results.

## 6. Conclusion

In summary, patients with SC, BD, and MDD have poorer cognitive and executive function than healthy individuals, even when these patients are in the stable state. The results of previous research on cognitive and executive deficits in SC, BD, and MDD are controversial. Some studies have reported similar deficits in SC and BD patients [51]. While many studies have pointed to more deficits in SC patients [50], a number of studies have also pointed to more defects in BD patients [11]. Overall, the results of the present study showed that cognitive deficits in MDD patients are more than SC and BD patients, but executive deficits in MDD patients are less than those in SC and BD patients. Also, cognitive and executive deficits in BD patients are more than those in SC patients. Assessment of cognitive and executive function in SC, BD, and MDD patients can help in understanding the pathology of these disorders.

## Figures and Tables

**Table 1:** Demographic and clinical characteristics of subjects by study groups.

Characteristics	SC (*N* = 64)	BD (*N* = 68)	MDD (*N* = 62)	HC (*N* = 75)	*t*, *F*, or **χ**^2^	*p*
Average age (years)	31.04 (6.01)	33.02 (7.03)	32.02 (5.08)	31.01 (7.08)	18.1^*F*^	0.687
Gender (M/F)	32/32	30/38	25/37	29/46	1.85^**χ**^2^^	0.362
Years of education	11.05 (2.02)	10.00 (2.01)	12.07 (2.04)	10.02 (2.07)	24.5^*F*^	0.142
BDI-II	25.05 (12.00)	9.00 (5.00)	28.06 (4.02)	7.04 (2.03)	82.5^*F*^	0.001
Age at first hospitalization (years)	23.06 (3.02)	25.01 (7.04)	24.06 (3.02)		2.1^*t*^	0.548
Number of hospitalization	2.01 (1.02)	3.07 (2.03)	2.09 (2.03)		0.3^**χ**^2^^	0.654
Comorbidity	29.58%	36.76%	34.65%		1.1^**χ**^2^^	0.027
PANSS-P	11.09 (3.06)					
PANSS-N	24.02 (4.08)					
YMRS		13.06 (7.04)				

*t*: *t* value; *F*: *F* value; *χ*^2^: chi-square value; YMRS: Young Mania Rating Scale; PANSS-P: Positive and Negative Schizophrenia Scale-Positive Section; PANSS-N: Positive and Negative Schizophrenia Scale-Negative Section.

**Table 2:** Results of the multivariate analysis of variance (MANOVA) in cognitive and executive function scales.

Variable	SC *n* = 64		BD *n* = 68		MDD *n* = 62		HC *n* = 75				
	*M*	SD	*M*	SD	*M*	SD	*M*	SD	*F*	*p*	Games-Howell
Cognitive scales											
SGIT	22.00	5.00	21.00	6.00	31.02	2.05	33.00	3.09	119.00	*p* ≤ 0.001	HC > SC&BD&MDD, MDD > SC&BD, BD < SC
TMT a	47.00	19.00	49.00	22.00	54.00	17.00	28.00	8.00	29.00	*p* ≤ 0.001	HC < SC&BD&MDD, MDD < SC&BD, SC < BD
TMT b	90.00	40.40	106.00	48.00	112.00	45.00	55.00	21.00	32.03	*p* ≤ 0.001	HC < SC&BD&MDD, MDD < SC&BD, SC < BD
Ruler	15.00	3.00	20.00	3.00	23.00	3.03	14.00	3.00	39.00	*p* ≤ 0.001	HC < SC&BD&MDD, MDD < SC&BD, SC < BD
RT time	1231.00	780.00	1413.00	320.00	1567.00	430.00	1132.00	245.00	7.00	0.001	HC < SC&BD&MDD, MDD < SC&BD, SC < BD
RT accuracy	68.00	32.00	91.00	5.00	93.00	4.00	96.00	3.00	42.00	*p* ≤ 0.001	HC > SC&BD&MDD, MDD > SC&BD, BD > SC
Executive scales											
TOL	20.00	7.00	12.00	6.00	31.00	5.00	37.00	11.00	121.00	*p* ≤ 0.001	HC > SC&BD&MDD, MDD > SC&BD, SC > BD
WCST											
Correct	70.00	21.00	49.00	12.02	72.00	17.00	75.00	18.00	36.00	*p* ≤ 0.001	HC > SC&BD&MDD, MDD > SC&BD, SC > BD
E-Per	9.00	6.00	40.00	14.00	8.00	4.00	29.00	13.00	58.00	*p* ≤ 0.001	BD > HC&MMD&SC, HC > SC&MDD, SC > MDD
E-N-Per	49.00	25.00	39.00	12.00	48.00	24.00	24.00	10.00	40.00	*p* ≤ 0.001	SC&MDD > BD&HC, BD > HC

^∗^ *p* < 0.01. SGIT: general intelligence; TMT a: Trial Making Test (a form); TMT b: Trial Making Test (b form); Ruler: reaction time; RT time: reaction time (time item); RT accuracy: reaction time (correct responses); WCST: Wisconsin Card Sorting Task; Correct: correct responses from WCST; E-Per: perseverative errors from WCST; E-N-Per: nonperseverative errors from WCST.

## Data Availability

The data used to support the findings of this study are included within the article.
